# Alterations in Cerebrospinal Fluid in Patients with Bipolar Syndromes

**DOI:** 10.3389/fpsyt.2016.00194

**Published:** 2016-12-08

**Authors:** Dominique Endres, Rick Dersch, Tilman Hottenrott, Evgeniy Perlov, Simon Maier, Dietrich van Calker, Benedikt Hochstuhl, Nils Venhoff, Oliver Stich, Ludger Tebartz van Elst

**Affiliations:** ^1^Section for Experimental Neuropsychiatry, Department for Psychiatry and Psychotherapy, University Medical Center Freiburg, Freiburg, Germany; ^2^Department for Neurology, University Medical Center Freiburg, Freiburg, Germany; ^3^Department for Psychiatry and Psychotherapy, University Medical Center Freiburg, Freiburg, Germany; ^4^Forensic Psychiatric Service, University of Bern, Bern, Switzerland; ^5^Department of Rheumatology and Clinical Immunology, University Medical Center Freiburg, Freiburg, Germany

**Keywords:** cerebrospinal fluid, bipolar disorder, blood–brain-barrier dysfunction, immunological encephalopathy, mild encephalitis

## Abstract

Bipolar disorder (BD) is a severe and lifelong condition. Primary endogenic polygenetic forms are common. Secondary organic forms have received increasing interest recently due to the detection of immunological encephalopathies that mimic various psychiatric syndromes, including BD. However, only limited data about routine findings of cerebrospinal fluid (CSF) analyses in BD are available. Therefore, we investigated the frequency of alterations in the CSF in patients with BD and the association with autoantibodies, cerebral magnetic resonance imaging, and electroencephalography findings. CSF samples of patients with BD collected from January 1998 until December 2015 were analyzed retrospectively. Patients with preexisting causes for alterations in the CSF (e.g., patients with obvious past or current neurological disorders) were excluded. In total, 63 patients with BD fulfilled the inclusion criteria for the study. In 1.6% of the patients with BD, an increased white blood cell count was found in the CSF. Increased albumin quotients were found in 12.9% of the patients, oligoclonal bands (OCBs) in 1.6%, and increased immunoglobulin (Ig) G indices in 3.2% (OCBs were not measured in case of increased IgG indices). No significant differences in CSF findings were found between patients with manic and depressive episodes. The main findings of this open uncontrolled study are that alterations in the CSF may be found in a small, but potentially relevant, subgroup of patients with BD. These findings are discussed in light of the new concepts of mild encephalitis and immunological encephalopathy. The detection of patients with possibly secondary organic bipolar syndromes could open up new causal treatment options with immunomodulatory medication.

## Introduction

### Bipolar Disorder

Bipolar disorder (BD) is a severe, lifelong condition with prevalence rates of 1–4% (1, 2). The underlying pathophysiological mechanisms are not completely understood. Primary polygenetic forms might be common (3). However, secondary forms due to inflammatory processes have received increased interest in the last few decades since autoantibody-associated immunological encephalopathies that mimic various psychiatric syndromes were detected (4). In line with this, we earlier published several case reports about patients with autoantibody-associated immunological encephalopathies who presented with isolated depressive or schizophreniform syndromes (5–8). Other authors have also described bipolar syndromes associated with underlying immunological encephalopathies [e.g., caused by thyroid autoantibodies (9–11)]. The idea of immunological dysfunction leading to mild encephalitis (12) is supported by epidemiological observations (i.e., increased rate of other autoimmune diseases in BD) and laboratory findings [e.g., circulating immune markers, inflammatory central nervous system (CNS) alterations (13)].

### Cerebrospinal Fluid Analyses and Earlier Findings in Bipolar Disorder

Cerebrospinal fluid (CSF) analysis is the most precise method for detecting CNS inflammation. In routine CSF analysis, the total white blood cell (WBC) count, total protein concentration, albumin quotient, intrathecal immunoglobulin synthesis (IgG/A/M), IgG index, oligoclonal bands (OCBs), and lactate concentration are measured (14). Reports of routine CSF findings in patients with BD are scarce and have limited sample size. Pazzaglia et al. described increased total protein concentration in 55% of male patients with BD compared to only 10.5% in female patients with BD (15). This gender-specific finding was confirmed in another study (16). However, increased total protein concentrations are an unspecific and imprecise marker for blood–brain barrier (BBB) function, which is assessed more precisely by the albumin quotient (14). BBB dysfunction might be due to antipsychotic treatment in patients with BD (17). Elevated lactate concentrations are typically found in patients with purulent meningitis but also in patients with ischemic stroke and mitochondriopathies (14). Interestingly, Regenold et al. (18) also found increased lactate concentrations in 5 out of 15 patients with BD. The researchers interpreted this finding as a consequence of mitochondrial dysfunction (18).

### Rationale of the Study

There is relevant evidence that inflammatory processes might lead to secondary forms of BD. However, only limited data about CSF basic diagnostic findings in BD are available. The current retrospective study was performed within this context. The aim of this project was to investigate the frequency of alterations in the CSF in BD. It was hypothesized that alterations would be found in the WBC count and BBB function, as well as that intrathecal humoral immune response would be found in the CSF of bipolar patient subgroups as markers of immunological dysbalance. In addition, CSF findings for patients with manic and depressive episodes were compared in an exploratory manner to detect possible differences.

## Participants and Methods

The study was approved by the local ethics committee (Faculty of Medicine, Freiburg University, EK-Fr 609/14). All measurements were part of the routine clinical workup. All patients gave written informed consent for lumbar punctures and cerebral magnetic resonance imaging (cMRI) diagnostics.

### CSF Samples

The CSF samples collected from January 1998 until December 2015 were analyzed retrospectively. The information was extracted from our CSF database, which was established in previous projects (19, 20). Routine CSF results were available for 75 patients with BD. Some of the patients were included in a previous research project (21). Comorbid somatic, neurological, and psychiatric diseases were obtained from medical reports. Patients with obvious past or current brain disorders, such as infectious encephalitis or meningitis, demyelinating diseases, stroke, brain tumors, epilepsy, delirium, dementia, traumatic brain disease, or earlier brain surgery were excluded as were hemolytic CSF samples. Headache due to migraine or tension headache and mild cognitive impairment were not exclusion criteria. Finally, 63 patients with BD were included in the descriptive and statistical analyses (Tables 1 and 2).

**Table 1:** **The bipolar disorder patient cohort**.

Available bipolar disorder CSF cohort from 1998 until 2015	75
Reasons for exclusion	
Infectious encephalitis	2 (1× viral encephalitis, 1× tick-borne encephalitis)
Demyelinating diseases	2 (2× multiple sclerosis)
Delirium	1 (most likely medication-induced)
Dementia	2 (1× Alzheimer’s disease, 1× multifactorial dementia)
Brain tumor/brain surgery	1 (medulloblastoma)
Movement disorders	2 (1× Parkinson’s disease, 1× unclear spastic tetraparesis)
Traumatic brain injury	1 (severe cerebral contusion)
Previous stroke	1 (repeated ischemic stroke)

**Bipolar disorder cohort for analyses**	63


**Table 2:** **Available datasets**.

Diagnostic measurements	Number of samples
CSF basic diagnostics (WBC count, protein concentration, albumin quotient, and intrathecal immunoglobulin synthesis)	63
Intracellular synaptic and onconeural antigens (GAD, amphiphysin, Yo, Hu, Ri, Cv2/CRMP5, Ma1, Ma2, and SOX1)	29
Antibodies against neuronal cell surface antigens (NMDAR, AMPA-1/2-R, GABA-B-R, and VGKC-complex: LGI1 and CASPR2)	23
Electroencephalography data sets	61
Magnetic resonance imaging data sets	50^a^

*^a^For eight patients, only cranial computer tomography was available*.

*WBC, white blood cell; GAD, glutamic acid decarboxylase; Yo/Hu/Ri, abbreviations of first patients’ name; Cv2/CRMP5, anti-collapsin response-mediator protein; Ma1/Ma2, 37, and 40 kDa neuronal proteins; SOX1, sry-like high-mobility group box 1; NMDAR, N-methyl-d-aspartat-receptor; AMPA-1/2-R, α-amino-3-hydroxy-5-methyl-4-isoxazole-propionic acid receptor; GABA-B-R, γ-aminobutyric acid receptor; VGKC-complex, voltage-gated potassium channels complex; LGI1, leucine-rich glioma-inactivated 1; CASPR2, contactin-associated protein-like 2*.

### Procedure of Basic Diagnostic Analyses

Laboratory investigations were performed in the CSF laboratory of the Department of Neurology of the University Medical Center Freiburg.^1^ The immunological assessment methods were described in previous papers (19, 20). Briefly, CSF WBC count and cytological differentiation were performed with manual microscopy (Leica DMRB, Germany) using a Fuchs–Rosenthal counting chamber (Hecht-Assistant, Germany). The basic quantitative protein diagnostics included total CSF protein, albumin, and immunoglobulin G, M, and A concentrations in the CSF and serum (ProSpect System, Siemens, Erlangen, Germany). Paired CSF and serum samples collected on the same date were analyzed simultaneously. For detection of BBB dysfunction, age-related albumin quotients were calculated (22). Intrathecal immunoglobulin synthesis was considered significant if the intrathecal immunoglobulin fraction exceeded 10% of the Reibergram (22), the IgG index was >0.7 mg/l, and/or if the OCBs – measured using isoelectric focusing followed by immunofixation (Hydragel Isofocusing, Sebia, France) – were present exclusively or predominantly in the CSF (23). In cases of an increased IgG index, no OCBs were measured. To avoid false-positive findings, we also described the albumin quotient and the immunoglobulin fraction of the Reibergram in these cases. Clear-cut alterations in the CSF were defined as increased WBC count, increased age-dependent albumin quotient, or intrathecal immunoglobulin synthesis. Antineuronal antibodies against onconeural intracellular or synaptic antigens were analyzed using an immunoblot employing recombinant neuronal antigens as the substrate (ravo Diagnostika, Freiburg, Germany). CSF antibodies against neuronal cell surface antigens were measured using a non-specific cell-based [transfected human embryonic kidney (HEK) cells] indirect immunofluorescence assay (Euroimmun, Luebeck, Germany). The cMRI images were evaluated by experienced senior neuroradiologists, and the electroencephalograms (EEGs) were analyzed by in-house physicians.

### Statistical Analysis

All laboratory and instrument-based diagnostic findings, as well as clinical information, were entered into a Statistical Package for the Social Sciences (SPSS) database. The main findings of alterations were presented descriptively in absolute values and percentage figures. For group comparisons (for the CSF findings) between manic and depressive patients, an independent-sample *t*-test for continuous variables was performed. For group comparisons (for alterations in cMRI and EEG) between patients with and without alterations in the CSF, a Pearson’s two-sided chi-square test was performed. A *p* value of less than 0.05 was considered statistically significant for the statistical analyses.

## Results

### Demographic Data

The mean age of all patients was 48.35 ± 15.22 years. The gender ratio in the study was 25 men to 38 women. At the time of the lumbar puncture, the patient group comprised 18 patients with manic episodes, 35 with depressive episodes, and 7 with mixed episodes. In three patients, lumbar puncture was performed after recovery or remission of an acute manic or depressive episode; in all other patients, lumbar puncture was performed while the patients were symptomatic.

### Routine CSF Diagnostics

In the BD group, 1.6% showed an increased WBC count. A false-positive pleocytosis due to hemolytic CSF was excluded. In 40% of the samples, an increased total protein concentration and, in 12.9%, an increased albumin quotient was found. OCBs were detected in 1.6% and increased immunoglobulin (Ig) G indices in 3.2% (OCBs were not measured in case of increased IgG indices). The CSF findings are summarized in Table 3. The characteristics of the patients with CSF alterations are presented in Table 4. In Patient 3, the increased IgG index was in line with a normal albumin quotient and a relevant IgG fraction in the Reibergram (~50%). In Patient 4 (Table 4), the increased IgG index was found in combination with a normal albumin quotient and a borderline IgG fraction in the Reibergram (~10%). In summary, the basic CSF diagnostic findings showed alterations in 28 of the 63 patients with BD (44.4%). Apart from the increased total protein concentration, clear-cut alterations were found in 12 of 63 patients (19%). None of the patients with clear-cut CSF pathologies had combined alterations in WBC count, albumin quotient, and intrathecal immunoglobulin synthesis. No autoantibodies against intracellular antigens or neuronal cell surface antigens were found. Seven of the eight patients (87.5%) with increased albumin quotients were male. The EEG was abnormal in one of these eight patients (12.5%), and the cMRI showed pathologies in four out of six patients (66.7%).

**Table 3:** **CSF basic diagnostics in bipolar patients**.

CSF measurement	Mean ± SD	Number of cases	Frequency of alterations	Threshold
White blood cell count (*n* = 62)	1.35 ± 0.87	↔: 61; ↑: 1 (cell count: 6/μl)	1.6%	<5/μl
Total protein concentration (*n* = 60)	436.40 ± 218.93	↔: 36; ↑: 24	40%	<450 mg/l
Albumin quotient (*n* = 62)	5.97 ± 3.00	↔: 54; ↑: 8	12.9%	<40 years: 6.5 × 10^−3^; 40–60 years: 8.0 × 10^−3^; >60 years: 9.3 × 10^−3^ (14)
Immunoglobulin-G-index (*n* = 63)	0.50 ± 0.13	↔: 61; ↑: 2	3.2%	Immunoglobulin-G-index ≤0.7 mg/l (14)
Oligoclonal bands (*n* = 61^a^)	–	No: 58; Yes: 3 – OCB restricted to CSF: 1 – OCB mirror pattern: 2	4.9%; OCB restricted to CSF: 1.6%; OCB mirror pattern: 3.3%	No oligoclonal bands

*CSF, cerebrospinal fluid; OCBs, oligoclonal bands; ↔, value within normal range; ↑, value above normal upper limit of normal*.

*^a^In cases of increased IgG-Index, no oligoclonal bands were measured*.

**Table 4:** **Patients with increased WBC count, CSF-specific OCBs, or elevated IgG index**.

Nr.	Patient	CSF	cMRI	EEG	Neuropsych.	Other findings	Comment
1.	56 y, female, bipolar disorder, depressive episode	Increased WBC count (6 cells/μl), protein concentration slightly increased (510 mg/l)	Over years, unchanged left cerebellar cavernoma with associated developmental venous anomaly without hemorrhage; mild supratentorial atrophy and unspecific white matter lesions	Normal	Slight slowing in word fluency, retentiveness, and attention	No infectious causes (borreliosis, lues, etc.); autoantibody screening not performed	–
2.	21 y, female, bipolar disorder, depressive episode	CSF-specific oligoclonal bands	Alterations of the right lateral ventricle with mild gliosis and reduced white matter volume	Intermittent generalized slowing	Deficits in all domains of TAP	Increased thyreoglobulin antibodies; autoantibody screening negative (including rheumatological screening), the “MRZ” reaction was negative	No typical relapses suggestive for multiple sclerosis episodes were reported; the Swanton criteria for dissemination in time and space were not fulfilled
3.	61 y, female, bipolar disorder, depression	Increased IgG-index (1.3); IgG fraction in the Reibergram: ~50%	Mild increased CSF spaces, microangiopathic white matter lesions, the cMRI was affected by many artifacts	Normal	n.p.	Comorbid hypothyroidism (thyroid autoantibodies not measured) and psoriasis vulgaris, autoantibody screening not performed	No typical relapses suggestive for multiple sclerosis episodes were reported
4.	37 y, male, bipolar disorder, manic episode	Increased IgG-index (0.81); IgG fraction in the Reibergram: ~10%	Normal	Dysrhytmic EEG with β-overlap	n.p.	Earlier lues infection; autoantibody screening not performed	Elevated IgG index possibly due to clinically inapparent CNS infection after peripheral syphilis infection

*CSF, cerebrospinal fluid; cMRI, cerebral magnetic resonance imaging; EEG, electroencephalography; TAP, test for attentional performance; n.p., not performed; MRZ-reaction, polyspecific, intrathecal humoral immune response against measles, rubella, and varicella zoster virus; y, years; IgG, immunoglobulin G*.

### cMRI and EEG

In 60% of the patients, cMRI alterations were detected. In most cases, unspecific white matter lesions (44%) were detected. EEG abnormalities were found in 23% of the patients, the majority of which were intermittent generalized slow activities (IRDAs in 15%; Table 5).

**Table 5:** **cMRI and EEG pathologies in patients with bipolar syndromes**.

Localization of cMRI alterations^a^	Frequency absolute^b^ (%)
White matter lesions/cerebral microangiopathy	22/50 (44)
Cortical atrophy	2/50 (4)
Postischemic changes	1/50 (2)
Other alterations	3/50 (6)
Cerebellar cavernoma	1/50 (2)
Mild asymmetrical hippocampi	1/50 (2)
Mild gliosis	1/50 (2)
Anatomic variations	2/50 (4)
Arteriovenous malformation	1/50 (2)
Aplasia of the left transverse sinus	1/50 (2)
Overall cMRI alterations	30/50 (60)

**EEG pathologies**	**Frequency absolute^b^ (%)**

Continuous generalized slow activity	2/61 (3)
Continuous regional slow activity	0/61 (0)
Intermittent generalized slow activity	9/61 (15)
Intermittent regional slow activity	3/61 (5)
Epileptic activity	0/61 (0)
Overall EEG pathologies	14/61 (23)

*^a^For eight patients, only cranial computer tomography was available*.

*^b^Only the predominant cMRI lesion or EEG alteration is listed for each patient*.

*cMRI, cerebral magnetic resonance imaging; EEG, electroencephalography*.

### Subgroup Analyses

A comparison of the CSF findings in patients with manic (*n* = 18) and depressive symptoms (*n* = 35) showed no statistically significant differences in the WBC count (1.24 ± 0.44 vs. 1.40 ± 1.06; *p* = 0.543, *n* = 52), total protein concentration (382.50 ± 172.99 vs. 465.65 ± 255.47; *p* = 0.245, *n* = 50), albumin quotient (5.48 ± 2.48 vs. 6.36 ± 3.47; *p* = 0.350, *n* = 52), and IgG index (0.49 ± 0.09 vs. 0.50 ± 0.16; *p* = 0.838, *n* = 53). In addition, patients with (*n* = 12) and without (*n* = 51) clear-cut alterations in the CSF were compared. Alterations in the CSF were detected significantly more often in male patients (8 of 12 were male; chi-square = 4.5, *p* = 0.034). The cMRI pathologies (chi-square = 0.01, *p* = 0.904) and the rate of EEG alterations (chi-square = 0.33, *p* = 0.564) was not statistically different between the two groups.

## Discussion

As the main findings of this open, retrospective, uncontrolled study, we observed pathological alterations in the CSF in 19% of patients with BD. The CSF findings did not differ between manic and depressive patients in a statistically significant way. Male patients showed a higher rate of clear-cut alterations in the CSF.

### Limitations

The first main limitation of this retrospective study is the missing control group. Therefore, the current study’s findings could not be compared to those of healthy controls. However, due to the potential side effects of lumbar punctures (e.g., headache, vomiting, nerve damage, and infection), at the current stage of research, it was not ethically justifiable to puncture healthy controls. Therefore, we compared our findings with well-established reference values based on extensive work by Reiber et al.^2^ Furthermore, we compared our results to those for other psychiatric patient cohorts, which were analyzed with the same methods in the same laboratory (19, 20). In cases of increased IgG indices, OCBs were not measured earlier in our laboratory. However, the findings of increased IgG indices are susceptible [e.g., due to increased albumin quotients; (22)]. To avoid the production of unreliable results, we also looked at the albumin quotient and the IgG fraction in the Reibergrams. Further studies should always analyze OCBs. The second main limitation of this study is due to its open and retrospective nature. At the University Clinic of Freiburg, for many years, CSF analyses had been offered and generally was performed only in selected patients with clinical signs or constellations suggestive of organic features. This of course leads to a probable selection bias of the sample. Therefore, the findings cannot be generalized to the overall group of bipolar patients presenting to a psychiatric clinic. However, to avoid an overinclusion of patients with suggestive organic features in this cohort, all patients with comorbid disorders associated with alterations in the CSF were excluded (see Table 1). Thus, only patients without preexisting organic causes for secondary organic bipolar syndromes were analyzed, and thus, from a clinical perspective, a cohort of patients with apparently endogenic primary BD was created. Due to the retrospective approach, we were not able to analyze the role of infrequent possible extracranial-influencing factors, such as spinal canal stenosis. Because of the retrospective character and the limited number of patients, other potentially relevant factors like medication have not been adequately addressed in this study. This also led to an incomplete dataset (e.g., autoantibodies were not measured in all patients). Thus, bigger prospective studies should be performed with predefined measurement protocols, including analyses of medicated and unmedicated BD patients. In spite of these shortcomings, this study is clinically important because it is, to the authors’ knowledge, the first study that focused primarily on complete routine CSF findings in combination with cMRI and EEG findings. Previous studies mostly focused on the role of BBB dysfunction (15, 16).

### Results in the Context of Previous Studies

The results are in line with those of previous studies that described increased total protein concentrations in some patients with BD (15, 16). We also found CSF alterations predominantly in male patients (16). In comparison to patients with psychosis, the rate of alterations in the CSF was lower. In patients with psychosis, increased cell counts were found in 3.4% (vs. 1.6% in BD), an elevated albumin quotient in 21.8% (vs. 12.9% in BD), and augmented intrathecal immunoglobulin synthesis in 7.2% [vs. potentially 4.8% in BD; (19)]. These differences support the idea that bipolar syndromes occur only in a small subgroup due to secondary inflammatory processes. This observation relates well to the clinical presentation of intermittent full remission without causal (e.g., immunomodulatory and anti-infectious) therapy. One might speculate that secondary forms due to inflammatory or neurodegenerative processes lead to chronic courses of the disease if the forms are not treated causally. However, our findings of clear-cut alterations in the CSF in 19% of patients are compatible with mild encephalitis (12) and immunological encephalopathies (4) in defined subgroups of patients with BD. These patients often show unspecific alterations in the CSF. Patients with Hashimoto’s encephalopathy (HE) showed mild lymphocytic pleocytosis in 25% of cases, increased protein levels in 85%, and OCBs in 8.3% (24). Patients with anti-*N*-methyl-d-aspartic acid receptor (NMDAR) encephalitis showed initially abnormal findings in 80% of the cases. WBC counts were often mildly increased, and in 60% of patients, CSF-specific OCBs were detected (25). EEG alterations were found in 95% of patients with HE and nearly all patients with anti-NMDA encephalitis (4). In the cMRI of patients with HE, often normal or non-specific white matter lesions were detected (in 74%), and about 20% of the patients had diffuse increased signaling on T2-weight images (24). In 50% of patients with anti-NMDA-R encephalitis, the cMRI was unremarkable, and in the other half of the patients, T2 or FLAIR signal hyperintensity was found in the hippocampi, and in the cerebellar or cerebral cortex, frontobasal and insular regions, basal ganglia, and brainstem (25).

### Alterations in the CSF in the Current Study and in General

Increased WBC counts, intrathecal immunoglobulin synthesis, and increased albumin quotients are unspecific but noticeable CSF findings (22). Increased WBC counts and intrathecal immunoglobulin synthesis are associated with acute and chronic inflammatory processes. In contrast, increased albumin quotients are unspecific findings. They can be caused by a broad spectrum of inflammatory CNS processes but also by systemic disorders, such as alcohol or diabetes-induced polyneuropathy, as well as by spinal canal stenosis (14).

#### WBC Count

Slightly increased WBC counts (5–30 cells/μl) can be due to stimulus pleocytosis (e.g., epileptic seizure) and are often found in immunological encephalopathies (4, 14). WBC counts above 30/μl are increased significantly and are distinct signs of infectious neuroinflammation [i.e., neuroborreliosis, neurosyphilis, meningitis, brain abscess, etc. (14)]. One patient in our cohort (Patient 1, Table 4) showed an isolated mild increase in WBC count (6 cells/μl) without other specific alterations in the CSF and no evidence of infectious agents. cMRI showed an over-the-years unchanged left cerebellar cavernoma with an associated developmental venous anomaly without hemorrhage. Moreover, cMRI depicted mild supratentorial atrophy and unspecific white matter lesions. No epileptic seizures were reported. One might speculate that the combination of mild pleocytosis in combination with cerebral atrophy, white matter lesions, and clinically evident cognitive deficits might be associated with mild chronic immunological encephalopathy. Alternatively, the findings could be unspecific, especially because no increased intrathecal immunoglobulin synthesis was detected.

#### Intrathecal Immunoglobulin Synthesis

One female patient (Patient 2, Table 4), with a normal WBC count, intact BBB function, and normal IgG index, showed weak CSF-specific OCBs; serological analyses showed increased thyroglobulin antibodies. The cMRI showed alterations around the right lateral ventricle with mild gliosis and reduced white matter volume. The EEG showed generalized slowing; the test for attentional performance (TAP) showed deficits in all domains. These findings are compatible with multiple sclerosis (MS) or HE. However, a relapsing disease course suggestive for MS was not reported by the patient. Additionally, the cMRI Swanton criteria for dissemination in time and space were not fulfilled and the MRZ reaction, a polyspecific, intrathecal humoral immune response against neurotropic agents (measles, rubella, and varicella zoster virus), which is possibly the most specific available CSF marker for MS (26), was also negative. Therefore, we were not able to diagnose MS or an early stage of this disease although previous studies showed an association between BD and MS (27, 28). The OCBs could also be an “immunological scar” after a previous CNS infection; however, a history of previous CNS infections was negative. HE could also be a possible reason. HE can be diagnosed by exclusion if the patient (1) suffers from encephalopathy with psychiatric symptoms (hallucinations, etc.), seizures, myoclonus, etc.; (2) has subclinical or mildly overt thyroid disease; (3) shows maximal non-specific cMRI alterations; (4) shows the presence of serum thyroid antibodies; and (5) shows the absence of autoantibodies against intracellular and cell-surface antigens (29). However, no steroid treatment was performed in this patient, which might have supported this differential diagnosis (8). Patient 3, with an increased IgG index, normal albumin quotient, and significantly increased IgG fraction in the Reibergram (~50%) also showed atrophic alterations in the context of hypothyroidism. Clarification for Hashimoto’s thyroiditis was not performed, and perhaps, again, HE could be one possible reason. This patient also suffered from psoriasis vulgaris; therefore, an autoinflammatory predisposition seems to be plausible. Such associations with autoimmune diseases have been described previously (30). One might speculate that the bipolar symptoms and the thyroid and skin disease are different signs of immune dysregulation in this patient. In line with this, a previous case report described a patient with BD and psoriasis. During the depressive episodes, the skin lesions worsened, and during the manic episodes, the psoriasis improved without specific treatment (31). The increased IgG index in Patient 4 is associated with a normal albumin quotient and a borderline IgG fraction in the Reibergram (~10%). Alterations in this patient are most likely due to previous clinically inapparent syphilis infection of the CNS following the previous syphilis infection in the primary stage that was successfully treated with antibiotics. Unfortunately, a diagnostic workup for neurosyphilis (including calculation of the antibody index) and measuring of OCBs – which would have provided more clarity – were not available for this patient. Patients with depressive (in 5%) and manic syndromes (in 3%) have earlier been described as rare but possible clinical presentations of neurosyphilis (32, 33).

#### Albumin Quotient

The age-dependent serum and CSF albumin quotient is the reference standard for measuring BBB function (22). In a previous study we detected increased albumin quotients in 21.8% of patients with psychosis (19). In the present bipolar cohort the rate of patients with increased albumin quotients was 12.9% and, therefore, lower than in patients with psychosis but higher than in patients with other neurological disorders, like optic neuritis, where BBB dysfunction was only found in 3.8% (19, 34). Increased albumin quotients are unspecific but can also be found in most types of immunological encephalopathies. Some authors suggest that intermittent BBB dysfunction is required for the development of autoantibody-associated immunological encephalopathy. Following this line of thought, common autoantibodies in the serum [e.g., anti-NMDAR-antibodies (35, 36)] can reach the brain only in patients with BBB dysfunction (35).

### The Role of CSF Analyses in Patients with Bipolar Disorder

Currently, CSF analyses are not generally recommended in patients with bipolar syndromes according to present guidelines (e.g., Nice guidelines^3^; German S3-Praxisleitlinie^4^). To avoid overlooking clinically potentially important immunological and infectious encephalopathies in a previous paper, the authors suggested to consider CSF analyses for patients with (1) laboratory- (e.g., hyponatremia and thyroid autoantibodies) and instrument-based alterations (e.g., EEG and cMRI alterations), (2) (sub)acute beginning of the symptoms, (3) an association between the beginning of symptoms and a feverish condition or vegetative derailment, (4) atypical clinical presentation, and (5) additional neurological symptoms [e.g., epileptic seizures, myoclonic jerks, other movement disorders (4)].

## Conclusion

This open uncontrolled study revealed evidence for rare, but relevant, alterations in the CSF in patients with bipolar syndromes. Alterations in the CSF in patients with BD are less frequent than in patients with psychosis. In daily clinical practice, findings in MRI and EEG basic diagnostics, or an atypical clinical presentation (e.g., with neurological symptoms), should also lead the clinician to consider an underlying encephalopathic pathomechanism. Future studies should analyze CSF findings in a prospective, controlled setting and correct for frequent influential factors (e.g., alcohol use, medication, diabetes, age, and gender). The detection of patient subgroups with infectious or immunological encephalitis could open up new causal treatment options.

## Author Contributions

LTvE and DE initiated the study and conducted the data analyses. DE wrote the paper. DE and BH performed the data collection. All of the authors were crucially involved in the theoretical discussion and performance of this study. Furthermore, all of the authors read and approved the final version of this manuscript.

## Conflict of Interest Statement

DE and RD: none. TH: travel grants from Bayer Vital GmbH and Novartis. EP, SM, DC, and BH: none. NV: advisory boards, lectures, research, or travel grants within the last 3 years: Janssen-Cilag, Roche, Novartis, AbbVie, GSK, Medac, and Pfizer. OS: consulting and lecture fees, grant and research support from Bayer Vital GmbH, Biogen Idec, Genzyme, Merck Serono, Novartis, Sanofi-Aventis, and Teva. LTvE: advisory boards, lectures, or travel grants within the last 3 years: Eli Lilly, Medice, Novartis, Shire, UCB, GSK, Servier, Janssen, and Cyberonics.

## Footnotes

^1^www.uniklinik-freiburg.de/neurologie/klinik/diagnostische-einrichtungen/liquor-labor.html.

^2^www.horeiber.de/liquor.html.

^3^http://www.nice.org.uk.

^4^www.dgppn.de.
